# Understanding the Geography of COVID-19 Case Fatality Rates in China: A Spatial Autoregressive Probit-Log Linear Hurdle Analysis

**DOI:** 10.3389/fpubh.2022.751768

**Published:** 2022-02-15

**Authors:** Hanchen Yu, Xin Lao, Hengyu Gu, Zhihao Zhao, Honghao He

**Affiliations:** ^1^Center for Geographic Analysis, Harvard University, Cambridge, MA, United States; ^2^School of Economics and Management, China University of Geosciences, Beijing, China; ^3^Department of Geography and Resource Management, The Chinese University of Hong Kong, Hong Kong, China; ^4^School of Software and Microelectronics, Peking University, Beijing, China

**Keywords:** COVID-19, case fatality rate, spatial autocorrelation, spatial heterogeneity, hurdle model

## Abstract

This study employs a spatial autoregressive probit-log linear (SAP-Log) hurdle model to investigate the influencing factors on the probability of death and case fatality rate (CFR) of coronavirus disease 2019 (COVID-19) at the city level in China. The results demonstrate that the probability of death from COVID-19 and the CFR level are 2 different processes with different determinants. The number of confirmed cases and the number of doctors are closely associated with the death probability and CFR, and there exist differences in the CFR and its determinants between cities within Hubei Province and outside Hubei Province. The spatial probit model also presents positive spatial autocorrelation in death probabilities. It is worth noting that the medical resource sharing among cities and enjoyment of free medical treatment services of citizens makes China different from other countries. This study contributes to the growing literature on determinants of CFR with COVID-19 and has significant practical implications.

## Introduction

The ongoing coronavirus disease 2019 (COVID-19), as a rapidly spreading global pandemic, comes as a big blow to the economic and social development of the world and has become a global health concern. Case fatality rate (CFR), known as the proportion of deaths from a kind of disease to the number of confirmed cases of this disease (the proportion of infected people who die), is an important indicator to measure the severity degree of the epidemic (1), as well as a reflection of the government capacity to prevent and control the epidemic (2). Outbreaking in Wuhan, China, in January 2020, the virus rapidly spreads through Hubei Province and the rest of China. It then became under control within 2 months through stringent prevention and control measures taken by Chinese governments such as lockdown, wearing face masks, self-quarantine, the detection and isolation of infected individuals, contact-tracing, social distancing, traffic restrictions, and community containment (3). COVID-19, however, is now affecting countries all over the world. As of January 25, 2021, there are 98,977,480 confirmed cases and 2,126,232 deaths worldwide, covering 224 countries and regions. Therefore, it is quite necessary to investigate determinants on COVID-19 CFRs of cities in China. The experience of China in controlling the spread of the virus and reducing mortality can help to inform other countries to better cope with the local epidemic outbreaks.

The fatality rate of COVID-19 is affected by multiple factors, including air pollution, climatic conditions, demographic characteristics, socioeconomic factors, and the controlling measures. Many scholars focus on the close association between air pollution and COVID-19 cases and mortality rates (4–10). As an essential environmental factor, climatic conditions also influence the death rates of COVID-19 (11), mainly measured by temperature and air humidity (12–14). Demographic characteristics have remarkable effects on the mortality of patients with COVID-19: age is the dominant factor; besides, gender, race, ethnicity, medical history (such as comorbidity and obesity), and neighborhood characteristics also play a significant role in determining the CFR (15–19). The socioeconomic factors exert specific impacts on COVID-19 spread, including income, unemployment, inequality, poverty, total population, population density, human mobility, and medical resources (17, 19–22). Finally, the government actions (such as containment measures, travel restrictions, and social distancing policies) prove to be effective in mitigating the spread of the disease and reducing the confirmed cases and deaths (23–25).

Scholars employ traditional statistical methods (such as multivariate and panel regression) to reveal the effects of demographic and clinical characteristics on the mortality of patients with COVID-19 (26–29). At the regional level, scholars use GIS (Geographic information system)-based spatial analysis methods and spatial regression models to evaluate the impacts of environmental conditions, socioeconomic factors, demographic features (age, sexual, racial, and ethnic structure) on the spatial distribution of COVID-19 cases and deaths, based on the data of country level, state level, county level, or city level (16, 17, 21, 23, 30, 31).

The CFR of COVID-19 in China (4.79% on January 25, 2021) is more than the double of the worldwide CFR (2.15% on January 25, 2021). One of the reasons why CFR of China is so high may be that people knew little about the virus early in the epidemic. The studies on the determinants of COVID-19 mortality of China focus more on the individual level from the perspective of patients in Wuhan of Hubei Province (29, 32–34). There are relatively few studies at the city level in China, which mainly concern the impacts of air pollution, climatic factors, and medical resources (35–37). It is significant to analyze the influence factors at the city level, as Chinese city governments have played an important role in taking timely measures to mitigate the spread of the epidemic. Besides environmental factors and medical factors, demographic characteristics and socioeconomic factors also need to be considered. Since China has successfully controlled the spread of the virus by March 13, 2020, the CFRs of COVID-19 in many cities outside Hubei Province are zero, and the zero-inflated models will better fit the data (38, 39). As China is a large country with a vast territory, there inevitably exist spatial heterogeneity and spatial spillover effects among cities. Therefore, this study employs a spatial autoregressive probit-log (SAP-Log) linear hurdle model, a combination of zero-inflated models and spatial effects, to examine the determinants on COVID-19 CFRs of cities in China and thus provides evidence for responding to the public health crisis in the future. Specifically, we will address the following questions:

(1) Do the probability of death from COVID-19 and the CFR level belong to two different processes?
(2) If they are different processes, what are the respective determinants on them?
(3) Is there a significant spatial spillover effect in the CFR?
(4) Are there differences in the determinants of the CFR between cities within and outside Hubei Province?

## Methodology And Data

### Data Collection

The data of COVID-19 cumulative confirmed cases and deaths are collected from the China National Health Commissions (CNHCs) (http://www.nhc.gov.cn) and the provincial Health Commissions by March 13, 2020 and January 25, 2021. The dataset covered 280 prefecture-level cities that have public data online. The CFR (CFR-spring and CFR-2021) of COVID-19, as the dependent variable in this study, is measured by the number of deaths per 100 confirmed COVID-19 cases by March 13, 2020 and January 25, 2021, respectively. The cumulative CFR on March 13, 2020 and January 25, 2021, respectively, represents the first and second waves of COVID-19 spread. In the first wave of a massive disease outbreak, Chinese governments have no experience in dealing with this epidemic, and it takes 2 months to control the spread of the virus; while in the second wave, Chinese governments have enough experience in prevention and control measures, contributing to the rapid containment of sporadic outbreaks. The comparison of the confirmed cases and CFR between these 2 research periods can better reveal the influence factors on CFR in the whole process of responding to COVID-19 and the effect of disease controlling experience. When the pandemic is still ongoing, the current CFR will not reflect the real situation because the infected people are likely to die in the future. Until March 13, 2020, however, the first wave of epidemic spread has been curbed in China, and the confirmed cases and deaths do not grow considerably. Between March 13, 2020 and January 25, 2021, the confirmed cases with COVID-19 have grown very slowly, not to mention the CFR. Therefore, the CFR is a reasonable indicator to measure the developing state of the epidemic. The CFR by March 13, 2020 varies significantly from city to city, with 65 non-zero CFR and 215 zero CFR. By January 25, 2021, the number of cities with non-zero CFR has increased to 68.

**Figure 1:**
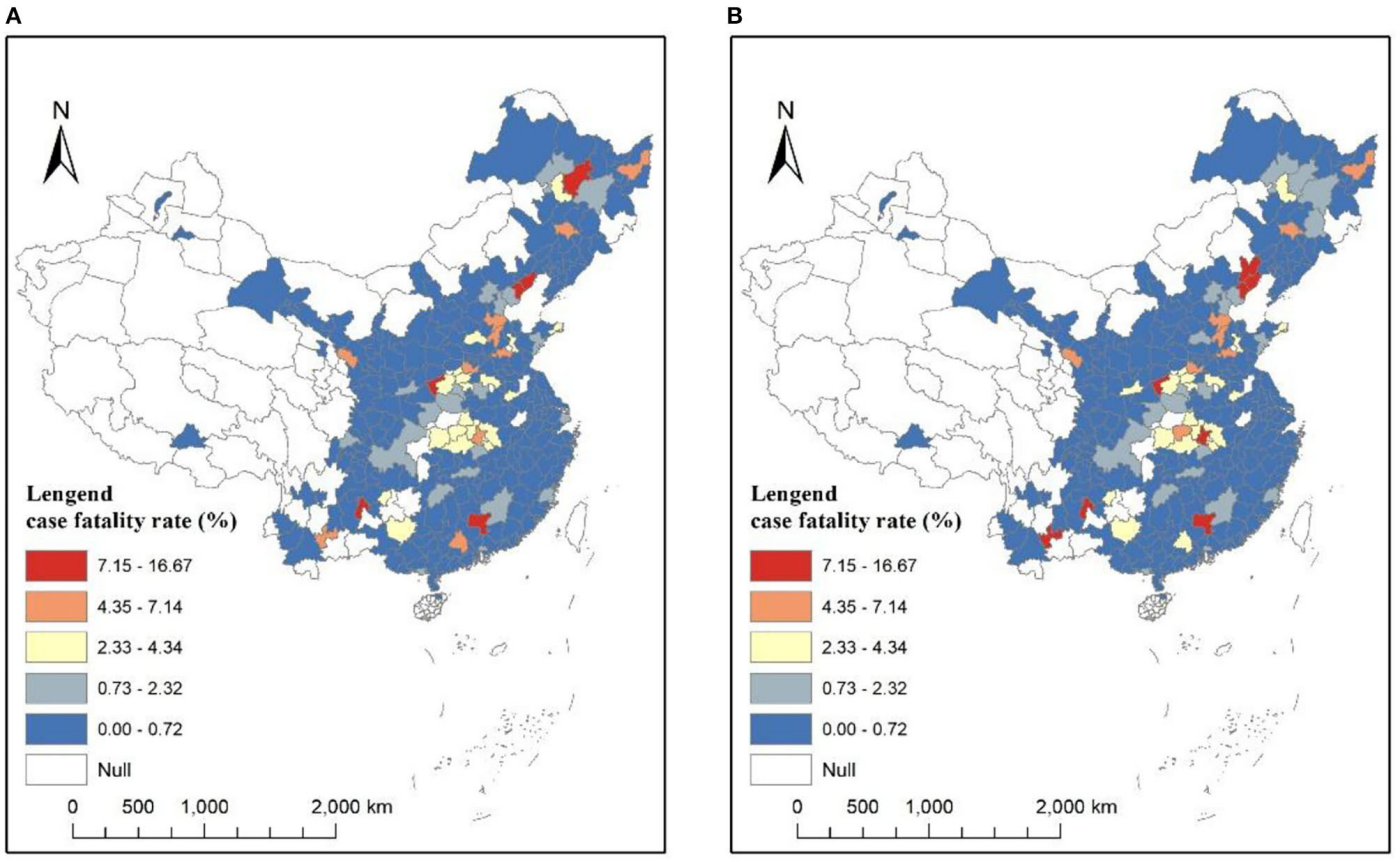
Coronavirus disease 2019 (COVID-19) case fatality rates (CFRs) at the city level. **(A)** March 13, 2020. **(B)** January 25, 2021.

**Figure 2:**
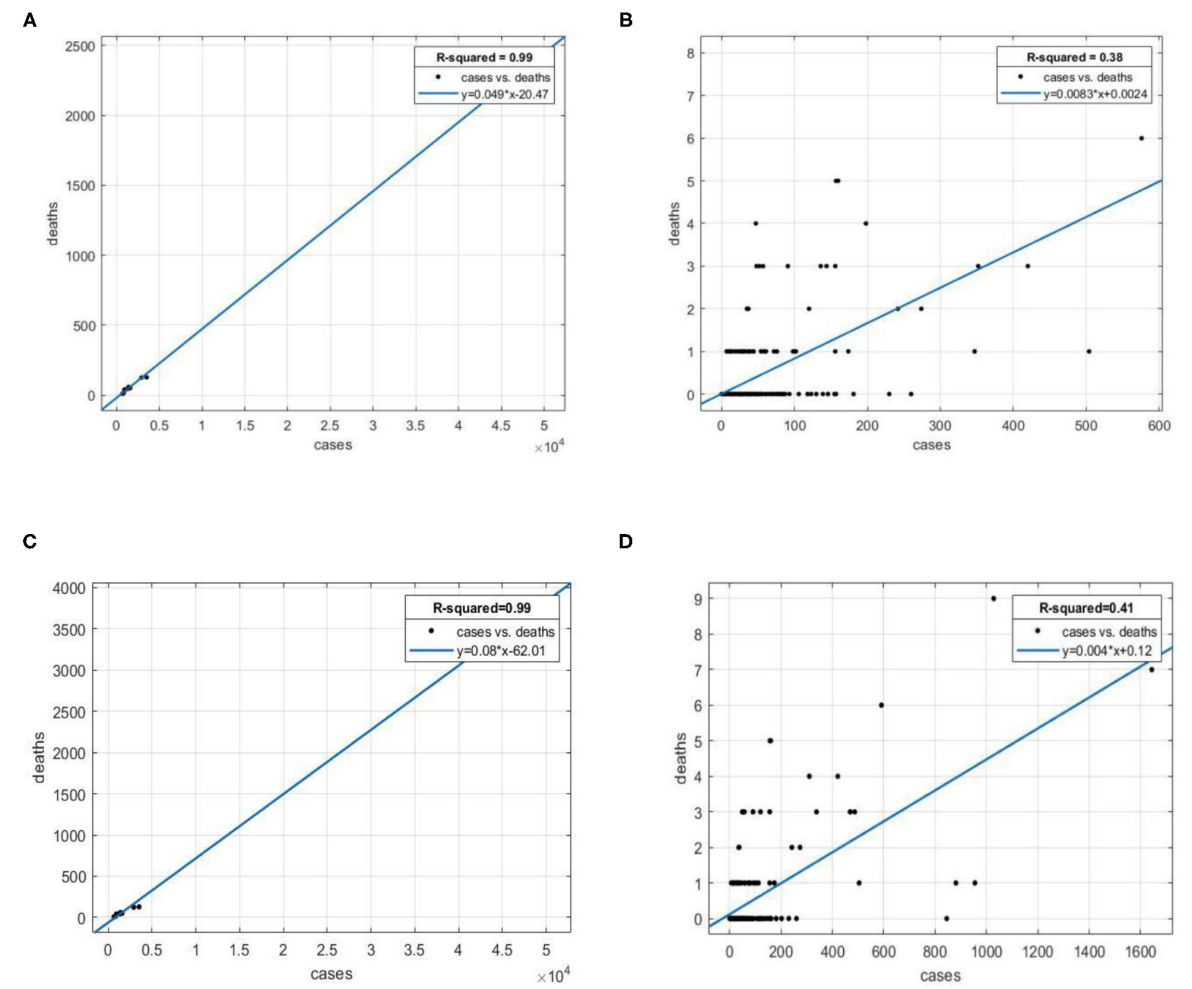
Scatterplot of COVID-19 deaths against cases. **(A)** Hubei Province, March 13, 2020. **(B)** Outside Hubei Province, March 13, 2020. **(C)** Hubei Province, January 25, 2021. **(D)** Outside Hubei Province, January 25, 2021.

The spatial distributions of COVID-19 CFRs are shown in Figure 1. As shown in Figures 1A,B, the distributions at both the times are similar. There exists significant spatial autocorrelation in CFR. The highest CFR values are mainly concentrated in Hubei Province cities, ranging from 2 to 7% on March 13, 2020. The spatial distribution of CFR in cities outside Hubei Province is relatively random and fluctuates considerably, ranging from 0 to 15%, due to a greater uncertainty of statistical inference caused by a smaller number of deaths. The confirmed cases in those cities are relatively smaller (even single digits), easily leading to extremely high CFR values (as shown in several spots of red color in Figure 1). COVID-19 cases vs. deaths in Hubei Province and other provinces are shown in Figure 2. The slope in Figure 2 represents the average CFR. The average CFR in Hubei Province has increased from 4.9 to 8.0%, while it decreased from 0.83 to 0.4% in other provinces. Cities in Hubei Province had much more cases and much higher CFR than other cities. There exists a significant linear relationship between the number of confirmed cases and death cases with a high value of *R*^2^ in Hubei Province (Figure 2A), illustrating that the CFR of each city in Hubei Province is consistent. The scatterplot of cities outside Hubei Province presents a roughly linear relationship, whereas a great disturbance on CFR emerges, resulting from the death cases ranging from 0 to 6.

### Statistical Analysis

Since the numbers of deaths in 215 out of 280 Chinese cities are zero in 2010, there exists an obvious zero-inflation problem in the regression of the CFR. However, extant studies on the CFR often ignored the zero-inflation problem, which led to statistical biases (38, 39). There are two reasons for the zero-inflation problem:

(1) The epidemic in China was under control. In total, 84% of the cases occurred in Hubei Province until March 13, 2020. The average confirmed cases inside and outside Hubei Province were 5,916.6 and 46.3, respectively. In contrast, there were fewer cases distributed in other regions. The slope in Figure 2B represents the CFR, which is 0.83%. Hence, the average number of deaths in those cities was 46.3 × 0.83% = 0.38. The average number of deaths was <1, resulting in no deaths in most cities.

(2) Some cities did not have the medical conditions to receive critically ill patients. Many critically ill patients were sent to surrounding cities with better medical resources.

A hurdle model is employed in this research to deal with the zero-inflation problem. It is a two-part model that specifies one process for zero counts and another process for positive counts.

The first part we used is a SAP model, which estimates the probability of attaining non-zero CFR predictors. The second part we used is a log-linear model, which estimates the predictors of the non-zero CFR values. Therefore, the SAP-Log hurdle model employed in this study is demonstrated as follows:

The SAP model part:


(1) $${\text{Y}}^{*}=\text{ρ}{\text{WY}}^{*}+\text{Xβ}+\varepsilon$$

The log-linear part:


(2) In(Y)=Xβ+ε

where Y^*^ signifies the probability of whether there is a death case in a city, assigned as 1 when the answer is yes, otherwise 0; Y denotes the CFR >0; X is the matrix of predictor variables, β is the parameter vector, ε is the error term. Medical, environmental, demographic, socioeconomic, and time factors were compiled and considered explanatory variables in Table 1.

**Table 1:** Description of explanatory variables.

**Category**	**Variable name**	**Description**	**Data sources**
Medical factors	Cases-spring	Cumulative number of confirmed COVID-19 cases by 13 March 2020	National Health Commission and the Provincial Health Commissions
	Cases-2021	Cumulative number of confirmed COVID-19 cases by 25 January 2021	National Health Commission and the Provincial Health Commissions
	Doctors	Number of doctors (10,000 doctors)	China City Statistical Yearbook 2019
Environmental factors	AQI-spring	Average Air Quality Index from 1 January 2020 to 13 March 2020	Ministry of Ecology and Environment of People's Republic of China
	AQI-2020	Average Air Quality Index in 2020	Ministry of Ecology and Environment of People's Republic of China
	Humidity-spring	Average humidity from 1 January 2020 to 13 March 2020 (%)	China Meteorological Administration
	Humidity-2020	Average humidity in 2020 (%)	China Meteorological Administration
	Temperature-spring	Average temperature from 1 January 2020 to 13 March 2020 (Celsius)	China Meteorological Administration
	Temperature-2020	Average temperature in 2020 (Celsius)	China Meteorological Administration
Demographic characteristics	Age	Average age of residents	Sixth National Population Census of China
	Ethnicity	Proportion of ethnic minorities (%)	Sixth National Population Census of China
	Gender	Percentage of males (%)	Sixth National Population Census of China
	Education	Average years of education (years)	Sixth National Population Census of China
	Household	Average number of persons per household	Sixth National Population Census of China
	Rurality	Percentage of rural population(%)	Sixth National Population Census of China
Socioeconomic factors	Insurance	Percentage of employees joining the urban basic medical care system (%)	China City Statistical Yearbook 2019
	Unemployment	Percentage of unemployment (%)	China City Statistical Yearbook 2019
	Wage	Average wage of employed staff and workers (10,000 yuan)	China City Statistical Yearbook 2019
	Poverty	Percentage of the population below poverty level (%)	China Rural Poverty Monitoring Report 2020
	Public transportation	Bus passenger volume per capita(Number of times)	China City Statistical Yearbook 2019
Time factor	First case	The number of days from the beginning of the epidemic to the first confirmed case.	National Health Commission and the Provincial Health Commissions

Based on the extant literature, the influence factors on the CFR with COVID-19 consist of medical factors, environmental factors, demographic characteristics, and socioeconomic factors. The medical factors are directly connected with the CFR, including the number of confirmed COVID-19 cases and the number of doctors. The former indicator is gathered from the National Health Commission and the Provincial Health Commissions, closely associated with the CFR as shown in Figure 2. The latter indicator is a good proxy to assess the healthcare capacity (medical resource availability and accessibility), explaining different mortality rates in different regions (40). The environmental factors are very important in determining the transmission capacity and pathogenicity of the virus, thus affecting the CFR (4–14), which consist of air quality (or pollution), humidity, and temperature. First, the particles of air pollution act as carriers for virus transmission (41), which aggravates the epidemic spread; second, people exposed to the ambient air pollution are often in a state of subhealth and easy to be infected with the virus (42); third, the air pollution will make the diseases of infected patients deteriorate, thus increasing the CFR (43). Air quality index (AQI) measures the degree of air pollution, including the contents of sulfur dioxide, nitrogen dioxide, carbon monoxide, ozone and particle matters, and it is commonly believed that air pollution emerges when the AQI is higher than 100. The demographic characteristics are composed of age, ethnicity, gender structures, average years of education, the average number of persons per household, and rurality, which signify different health conditions, living conditions, and capacities to respond to COVID-19 of the susceptible population, thus directly influencing COVID-19 mortality (15–19). The socioeconomic factors also matter in affecting the CFR, which incorporate the insurance coverage, the percentage of unemployment, the average wage of employed staff and workers, poverty, and public transportation. This kind of factors reflect the socioeconomic status of people and concern the living standards, sanitary conditions, and capacities to afford healthcare, thus exerting impacts on their health outcomes (17, 19–22). Regarding the specific data sources, the number of doctors (doctors), the percentage of unemployment (unemployment), the average wage of employed staff and workers (wage), the percentage of employees joining the urban basic medical care system (insurance), and the bus passenger volume per capita (public transportation) are collected from China City Statistical Yearbook 2019, which records the newest available data of cities in 2018. The percentage of the population below the poverty level (Poverty) is gathered from China Rural Poverty Monitoring Report 2020. The AQI daily observation data are acquired from the Ministry of Ecology and Environment of the People's Republic of China, and the average daily values of each city during the 2 research periods (from January 1, 2020 to March 13, 2020, and from January 1, 2020 to January 1, 2021) are calculated. Similarly, the average humidity (humidity) and the average temperature (temperature) during the research period are gathered from the China Meteorological Administration. The average age of residents (age), the proportion of ethnic minorities (ethnicity), the percentage of males (gender), the average years of education (education), the average number of persons per household (household), and the percentage of the rural population (rurality) come from the Sixth National Population Census of China, which records the demographic data of 2010 and is the latest data available in public because China conducts national population census every 10 years. As the time of the event may affect the mortality rate, we also collected the number of days from the beginning of the epidemic to the first confirmed case (First case) in the city.

## Analysis Of Results

To test the multicollinearity in the regression model, we calculated the variance inflation factor (VIF) and found that the VIF value of each variable is <4, indicating that there is no multicollinearity in our model. A series of control variables have been incorporated into the model, and the dependent variable is lagged from the independent and control variables, thus reducing the possible endogeneity problem to some extent. The descriptive statistical analysis of all variables is shown in Table 2. In both parts of the hurdle models, we established the models with CFR in 2020 and 2021 as the dependent variables. The cases, AQI, humidity, and temperature variables are different in the 2 research periods, while others are constant. We employed the average AQI, humidity, and temperature from January 1, 2020 to March 13, 2020 (AQI-spring, humidity-spring, and temperature-spring) to model CFR-2020 and the yearly average AQI, humidity, and temperature in 2020 (AQI-2020, humidity-2020, and temperature-2020) to model CFR-2021.

**Table 2:** Descriptive statistical analysis.

**Variables**	**Mean**	**Min**	**Max**	**SD**
CFR-spring	0.81	0.00	14.29	1.99
CFR-2021	0.83	0.00	16.67	2.16
Cases-spring	276.91	0.00	49,995.00	3,000.03
Cases-2021	299.74	0.00	50,355.00	3,023.17
Doctors	1.20	0.10	10.94	1.21
AQI-spring	57.23	23.24	105.52	17.73
Humidity-spring	66.14	31.66	90.60	14.16
Temperature-spring	7.13	−7.82	23.48	6.58
AQI-2020	56.62	23.73	99.94	16.18
Humidity-2020	65.49	30.15	85.46	13.10
Temperature-2020	16.56	2.01	27.89	5.30
Age	35.87	29.92	43.13	2.48
Ethnicity	92.20	11.89	99.99	16.07
Gender	51.38	47.27	99.10	3.00
Education	8.96	11.71	6.55	0.83
Household	3.07	4.75	2.04	0.46
Rurality	71.82	39.11	91.79	12.01
Poverty	1.74	0.00	5.8	1.57
Insurance	0.73	0.17	10.32	0.65
Unemployment	2.38	0.19	12.42	1.45
Wage	7.14	14.98	3.89	1.45
Public transportation	49.00	0.00	582.24	67.75
First case	4.03	50.00	1.00	8.80

In the first part of the hurdle model, we established a SAP model and a traditional probit model to regress the death probability of March 13, 2020 and January 25, 2021, respectively, with the results displayed in Table 3. It explains whether a city has a death case with COVID-19.

**Table 3:** First part: Regression estimates of the probit models.

	**Probit model (CFR-2020)**	**Spatial probit model (CFR-2020)**	**Probit model (CFR-2021)**	**Spatial probit model (CFR-2021)**
**Explanatory variables**	**Coefficients**			
Cases-2020	0.008***	0.001***		
Cases-2021			0.004***	0.003***
Doctors	0.178	0.473***	0.375**	0.408***
AQI-spring	0.013	0.012		
Humidity-spring	0.020*	0.002**		
Temperature-spring	−0.015	−0.012		
AQI-2020			0.001	−0.001
Humidity-2020			0.002	0.007
Temperature-2020			−0.048	−0.057**
Age	−0.154	−0.12	−0.124	−0.088
Ethnicity	−0.010	0.007	−0.007	0.008
Gender	−0.165	−0.010	−0.097	−0.034
Education	0.517**	0.371***	0.443*	0.494***
Household	0.073	0.136	0.456	0.627**
Rurality	−0.004	−0.009	−0.012	−0.009
Poverty	−0.095	−0.080	0.043	0.053
Insurance	0.912	0.598	0.718	0.594
Unemployment	−0.075	−0.076	−0.061	0.049
Wage	−0.106	−0.188*	−0.177	−0.173*
Public transportation	−0.004	−0.001	−0.005	−0.004
First case	−0.048	−0.065***	−0.081	−0.073***
ρ		0.168**		0.114**
Pseudo R^2^	0.363	0.365	0.358	0.363
Number of observations	280	280	280	280

* *p < 0.1*;

** *p < 0.05*;

*** *p < 0.01*.

By comparing the spatial and the non-spatial models, it is evident that the value of pseudo *R*^2^ of the spatial model is higher than that of the non-spatial model, indicating better goodness of fit. The regression coefficient of the spatially lagged term ρ is also significant. Therefore, the SAP model is more suitable, because the neglect of the spatial effect will cause omitted variable bias.

In the 2 spatial models of different research periods, the regression coefficients of cases, doctors, education, wage, first case, and ρ are all significant. The number of confirmed cases (cases) with COVID-19 is an important determinant of the appearance of death, which often emerges after the number of death cases reaches a certain level. The regression coefficient of cases is 0.001 and 0.003 at different times, demonstrating that the probability of death will increase by 0.1 and 0.3% with 1 newly increased confirmed case. The number of confirmed cases (cases) reflects the demand for treatment of COVID-19. During the period of epidemic outbreak, as the number of confirmed cases increases, there are limited medical resources (experienced doctors, medical facilities, and medicines) in most cities, which cannot satisfy the increasing demand for treatment and thus lead to a growth in CFR. Since doctors are an indicator to measure the medical resource availability and accessibility, its relation with the death probability should be negative. However, the results of the spatial probit models show that better medical resources will cause a higher possibility of death. Such a seemingly contradictory phenomenon is due to the uneven distribution of medical resources in China, there is a lack of necessary medical facilities in most cities. When severe cases happen in the cities with fewer medical resources, the governments tend to transfer the patients to the surrounding cities with more medical resources. At the same time, the patients with serious conditions will also choose to seek treatment in a city with more medical resources. Consequently, the cities with more medical resources will have higher death probabilities, as they gather the severe patients from surrounding cities, while the cities with fewer medical resources will have lower death probabilities. The regression coefficient of doctors displays that the death probability will increase by 40.8–47.3% with the augment of 10,000 doctors. The variable education measures the average education attainment level, which determines the type of job mainly based on physical labor or mental labor. People engaged in physical labor often have a higher health level than those of mental labor, owing to the fact that the former group has more physical exercise than the latter. Hence, people in the region of higher education level tend to have poorer health than the region with lower education level, thus causing worse disease conditions and increasing the risk of death from COVID-19. The death probability will grow by 37.1–49.4% with the increase of 1 year of education. Wage exerts negative impacts on the appearance of death: the new odds of death will decrease by 17.3–18.8% compared with the original odds, in response to a rise of 1 unit in wage. People with a higher wage level are inclined to receive early medical treatment and choose better medical treatment, and the prompt treatment and high-quality medical care both decrease the possibility of death. The first case shows the time of discovering the first confirmed case in a city. The later the first case appears, the better preparation the city will have for responding to the epidemic, thus leading to a lower possibility of death. It is because a city where the first confirmed case emerges late can learn experience from other cities.

The spatial effect coefficient ρ in the 2 spatial models of different research periods is significantly positive, indicating an evident spatial autocorrelation in CFR: on one hand, the high–high agglomeration signifies that a city with a high death probability is surrounded by cities that also have a high possibility of death; on the other hand, the low–low agglomeration represents that a city with a low death probability is surrounded by cities that also have a low possibility of death. The former phenomenon is caused by the transfer of critical patients among cities. When the medical resources of a city are not enough for the critical patients, part of critical patients will be transferred to surrounding cities for treatment. The relative shortage of medical resources in the city and the number of critical patients exceeding the medical care capacity will both require the support of medical resources from surrounding cities. The latter phenomenon results from the distance decay effect of medical knowledge and experience. A city with rich medical care experience in COVID-19 treatment will benefit surrounding cities, thus reducing the likelihood of death from COVID-19, which will be further verified in the second part model. The medical resource sharing among neighboring cities also contributes to this.

In the spatial model of the first research period, Humidity is significantly positive at the 5% level, indicating that a higher humidity increases the likelihood of death occurring in the early days of the outbreaks. A higher humidity may contribute to the survival and transmission of the virus. In the spatial model of the second research period, Humidity no longer influences CFR, but temperature and household have a marked impact on CFR, stating that lower temperature and larger family size will lead to a higher death possibility with COVID-19. The probable reason is that lower temperature is favorable for the survival and transmission of the virus, and a larger family size indicates a higher chance of transmission between family members.

In the probit part of the hurdle model, the regression result of the first research period is very similar to that of the second research period, which proves the robustness of our results.

**Figure 3:**
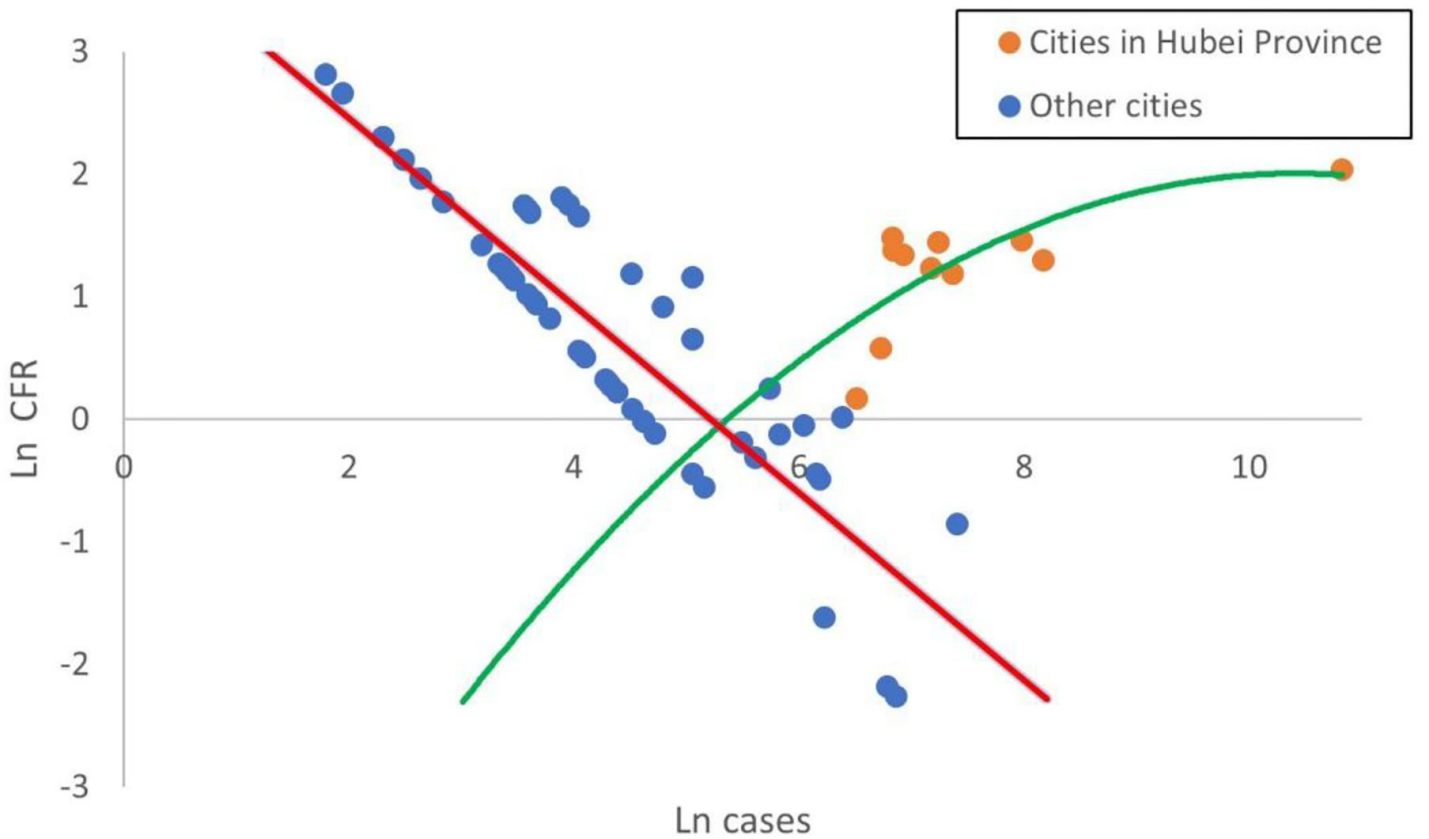
Scatterplot of COVID-19 ln CFR against ln cases.

Before the second part of the hurdle model, we first consider the relationship between Cases and CFR. Since the emergence of death has been explained by the first part model, we only analyze the cities that have death cases. Given the fact that the difference between cities is large, we take the logarithm of Cases and CFR and create a scatterplot in Figure 3.

Figure 3 demonstrates that there exist marked differences in the relationship between cities in Hubei Province and outside Hubei Province. There exists a negative linear relationship between ln CFR and ln cases in cities outside Hubei Province. This indicates that the medical treatment experience has a great impact on CFR in cities outside Hubei Province and the medical experience accumulation with the increasing confirmed cases will result in the CFR reduction. It is worth noting that some cities with relatively few confirmed cases even have higher CFR than Wuhan due to the lack of the medical experience. On the contrary, there exists a positive nonlinear relationship between ln CFR and ln Cases in cities within Hubei Province. This is mainly because the first outbreak of COVID-19 in Hubei Province in January 2020 caused an explosive growth in the number of confirmed cases. The limited medical resources in Hubei Province were overwhelmed and some critical patients could not get adequate treatment, giving rise to the increase of CFR along with cases. As cases grew, the CFR of Hubei Province reached a peak (e^∧^2) and then no longer increased anymore. Considering that there exist significant differences between the cities within and outside Hubei Province, i.e., spatial heterogeneity, we will build the regression models of cities within Hubei Province and outside Hubei Province, respectively.

The second part of the hurdle model also includes 2 cross-sections and considers the spatial heterogeneity of cities within and outside Hubei Province. The value of Moran's I is not significant, indicating no spatial effects. The regression results are shown in Table 4, with a relatively higher fitting degree (adj *R*^2^ > 0.7). First, we analyze the cities outside Hubei Province. The increase of 1% in the number of Ln Cases will cause CFR to decrease by 0.816 and 0.896% in 2020 and 2021, respectively, which implies that the accumulation of medical experience significantly reduces the CFR. Regarding the 11 cities within Hubei Province and considering the degree of freedom and multicollinearity of the model, we employ the stepwise regression method to select the variables. Cases, AQI and Humidity all have significant positive effects on CFR, while the other variables are non-significant in the model. The growth of the number of Cases quickly strains medical capacity and increases mortality. The environment also affects the CFR, with higher AQI and Humidity leading to higher CFR. A higher AQI means higher air pollution, which will contribute to the transmission of the virus and lower the resistance of people to diseases.

**Table 4:** Second part: Log-linear regression estimates for cities with nonzero case fatality rate (CFR).

	**Cities outside Hubei province: CFR-2020**	**Cities outside Hubei province: CFR-2021**	**Cities in Hubei province: CFR-2020**	**Cities in Hubei province: CFR-2021**
**Explanatory variables**	**Coefficients**			
Ln Cases-2020	−0.816***		0.250***	
Ln Cases-2021		−0.896***		0.347***
Doctors	0.125	0.147		
AQI-spring	0.009		0.056***	
Humidity-spring	−0.007		0.079***	
Temperature-spring	−0.021			
AQI-2020		0.016		0.077***
Humidity-2020		0.006		0.065***
Temperature-2020		−0.016		
Age	−0.015	−0.001		
Ethnicity	−0.004	0.001		
Gender	−0.004	0.060		
Education	−0.252	−0.287		
Household	−0.081	−0.129		
Rurality	−0.012	−0.014		
Poverty	−0.032	−0.019		
Insurance	−0.224	−0.346		
Unemployment	−0.056	−0.072		
Wage	−0.076	−0.031		
Public transportation	0.001	0.002		
First case	−0.007	−0.017		
Constant	8.965	4.756	−9.264***	−10.396***
Adj R^2^	0.714	0.771	0.717	0.885
Number of observations	54	57	11	11

* *p < 0.1*;

** *p < 0.05*;

*** *p < 0.01*.

## Conclusion And Discussion

Given the enormous damages to human society caused by the spread of COVID-19, robust scientific evidence will significantly contribute to the epidemic responses, especially the successful disease prevention and control experiences in China. Therefore, it is crucial to clarify the influence factors that significantly affect the CFR with COVID-19 by conducting a multicity study in China.

In this study, a SAP-Log hurdle model is employed to deal with the zero-inflation problem since nearly three quarters of cities have zero-value CFR, which dramatically reduces the estimation bias and improves the explanatory power and goodness of fit of the model. The hurdle model also reflects the two different processes: whether there is a death from COVID-19 in a city and how high the nonzero value of CFR in a city is. During these two different processes, the influence factors are different. The application of the hurdle model in the research on the CFR with COVID-19 will provide methodological guidance for epidemic response.

The main conclusions of this study are shown as follows:

First, the influencing factors on the death occurrence probability include the number of confirmed cases, the number of doctors, average humidity, average temperature, average years of education, the average wage of employed staff and workers, the average number of persons per household, the number of days from the beginning of the epidemic to the first confirmed case. The determinants of the CFR level include the number of confirmed cases, AQI, and average humidity. Regarding the determinants, the number of confirmed cases is the most significant variable in both the two parts of the hurdle model, which is much in evidence since it is the denominator of CFR. Cases have a positive impact on the death occurrence probability and the CFR level of cities within Hubei Province, but have a negative impact on the CFR level of cities outside Hubei Province, displaying a remarkable spatial heterogeneity. The former mechanism is that a larger number of confirmed cases will cause a higher death probability under a certain level of CFR. The latter mechanism is shown as below: In cities outside Hubei Province with relatively fewer confirmed cases, a larger number of confirmed cases will result in more abundant medical treatment experience and a lower CFR level; in cities within Hubei Province with relatively numerous confirmed cases, a larger number of confirmed cases will exert huge pressure on limited medical resources and cause higher CFR. Besides the number of confirmed cases, the number of doctors also exerts significant influences. Though cities with rich medical resources can provide better treatment, the transfer of critical patients from surrounding cities to these cities will drive up death probability. As it is known to all, air quality plays a vital role in the spread of COVID-19 because aerosol is a potential transmission route for COVID-19, embodied in the effects of AQI, humidity and temperature on CFR (44, 45). However, the other socioeconomic factors and demographic characteristics do not affect the CFR levels dramatically. The fact that people in China all enjoy free medical treatment services for COVID-19 significantly contributes to reduce CFR, no matter which levels of cities they are in and which kinds of groups they belong to.

Second, there exist significant spatial spillover effects in the death occurrence probabilities, i.e., a city where death from COVID-19 is likely to happen is surrounded by cities with higher death possibilities. The reasons for this are the transfer of critical patients among cities, the sharing of medical resources among cities, and the spatial spillover effects of medical knowledge dissemination.

The main findings of this study have certain policy implications: To begin with, given the importance of the number of confirmed cases in determining CFR, the proposal of “flatten the curve” (46) is still vital to help save lives and decrease CFR, by taking lockdown and social distancing measures to reduce the number of infected people in countries most affected by COVID-19, when limited by the current medical resources. Furthermore, the availability and accessibility of medical resources are very vital to control the spread of an epidemic, since the critical patients can be transferred to the large cities with better medical conditions in China, the medical treatment level of these large cities should be further improved. Considering the two different processes in the hurdle model, the governments should attach importance to the socioeconomic factors when preventing the emergence of death from this disease and lay much stress on the air quality when the aim is to reduce the CFR. In addition, the generally greater effects of the estimators in the model of the second research period than those in the first research period, as well as the significant effect of the number of days from the beginning of the epidemic to the first confirmed case on the death probability and CFR, have signified the influence of accumulated experiences in the response to COVID-19. The containment policies in China, including the immediate lockdown, community containment, self-quarantine, contact-tracing, as well as the free detection and medical treatment of this infectious disease for all residents, prove to be very effective in controlling the spread of the virus and reducing the CFR (47), which can also provide valuable references for other countries in the fight against the pandemic. Finally, the spatial probit models reveal the spatial spillover effects in the death probabilities, demonstrating that the popularization of medical knowledge and experience is very important to effectively control the spread of COVID-19. Hence, it is suggested that the city governments offer specialized training for the response to COVID-19.

Consistent with extant studies on COVID-19-related cases and deaths using spatial regression models in different countries (17, 19, 21, 48–51), this study also discovers that spatial regression models, with better goodness of fit than aspatial regression models, can partially explain the spatial heterogeneity in the CFR with COVID-19 and significantly reveal the spatial spillover effects on the CFR of neighboring cities and its various determinants (medical, environmental, demographic, and socioeconomic indicators). In addition, this study contributes to the existing literature by introducing a hurdle model into the spatial autoregressive model to deal with the zero-inflation problem, thus discovering two different processes influencing COVID-19 related deaths: whether there is a death from COVID-19 in a city and how high the non-zero value of CFR in a city is. There are relatively few studies on the determinants of COVID-19 confirmed cases and deaths in China employing spatial regression models, which focus more on COVID-19 transmission and the influencing factors on the number of COVID-19 cases (52–54), while this study considers the determinants on CFR with COVID-19 from different perspectives.

Despite its methodological contributions and practical implications, there still exist some deficiencies in this study. The dependent variable reflects the confirmed cases and CFR on March 13, 2020 and January 25, 2021, while some explanatory variables only reflect the annual averages limited by the data accessibility. What is more, among explanatory variables, the city-level socioeconomic data from *China City Statistical Yearbook 2019* records the situation of 2018, and the demographic data of Sixth National Population Census's record the conditions of 2010, which are all the most recent data available publicly in China. The policy-related variables (such as lockdowns, containment measures, travel restrictions, and social distancing policies) and hospitalization variables also play an important role in affecting CFR. However, the related data of each city is not available in China, so we cannot consider the effects of them in the model. Finally, the biological differences of residents among different cities can be taken into consideration in the future studies on determinants on the CFR with COVID-19 at the city level, including the effects of digit ratio (2D:4D) and ACE (Angiotensin-Converting Enzyme) I/D polymorphism on the CFR (55–58).

## Data Availability Statement

Publicly available datasets were analyzed in this study. This data can be found here: https://figshare.com/s/853348383d39f65bb6a6.

## Author Contributions

HY contributed to idea formulation, study design, methodology, data collection, and analysis. XL contributed to data interpretation and manuscript writing. HG contributed to discussion, review, and editing of writing. ZZ contributed to the collection of literature and data. HH contributed to the data collection. All authors contributed to the article and approved the submitted version.

## Funding

The corresponding author would like to acknowledge the financial support from the grants of National Natural Science Foundation of China (No. 42101226). HY would like to acknowledge the financial support for the grants of US National Science Foundation (Nos. 1841403, 1758786, and 2117455), the China Data Institute, and the Future Data Lab.

## Conflict of Interest

The authors declare that the research was conducted in the absence of any commercial or financial relationships that could be construed as a potential conflict of interest.

## Publisher's Note

All claims expressed in this article are solely those of the authors and do not necessarily represent those of their affiliated organizations, or those of the publisher, the editors and the reviewers. Any product that may be evaluated in this article, or claim that may be made by its manufacturer, is not guaranteed or endorsed by the publisher.
